# Effect of Chitosan and Hyperbranched Poly-L-Lysine Treatment on Quality of Cucumber (*Cucumis sativus* L.) during Storage

**DOI:** 10.3390/foods13091354

**Published:** 2024-04-27

**Authors:** Jianrui Sun, Jinglan Li, Ruirui Ren, Linlin Yao, Li Tong, Jiangfeng Yuan, Dahong Wang

**Affiliations:** College of Food and Bioengineering, Henan Engineering Research Center of Food Microbiology, Henan University of Science and Technology, Luoyang 471023, China; lijinglan1207@163.com (J.L.); 15090320603@163.com (R.R.); linlinyao1121@163.com (L.Y.); 15236675557@163.com (L.T.); jiangfengyuan@163.com (J.Y.); wangdahong2003@163.com (D.W.)

**Keywords:** cucumber, chitosan, hyperbranched poly-L-lysine, preservation

## Abstract

To enhance the storage time of cucumbers, this research investigated the impact of chitosan (CS) and hyperbranched poly-L-lysine (HBPL) on the quality and nutritional attributes of cucumbers when stored at a temperature of 25 °C. The results demonstrated that sensory evaluation scores for cucumbers treated with a CS–HBPL combination were significantly higher than the control (CK), CS, and HBPL groups. On the 18th day of storage, cucumbers in the CK group exhibited significant decay and softening; however, there was a decrease in hardness observed in the CS–HBPL group and no decay or noticeable sour taste was detected. Furthermore, compared to the CK group, treatment with CS–HBPL effectively delayed cucumber decay and weight loss rate while significantly inhibiting decreases in cucumber hardness and growth of surface microorganisms. Additionally, it substantially reduced losses of soluble protein content as well as vitamin C (Vc), reducing sugars, and total phenolic compounds within cucumbers, which were 4.7 mg/g, 4.7 mg/g, 0.94 mg/g, and 0.52 mg/kg, respectively. Moreover, compared to the CK group, combined treatment with CS–HBPL significantly inhibited malondialdehyde (MDA) accumulation and reducing relative electrolyte permeability within cucumbers, which were 1.45 μmol·g^−1^FW and 29.82%. Furthermore, it notably enhanced activities of superoxide dismutase (SOD) and catalase (CAT), while exerting a significant inhibitory effect on polyphenol oxidase (PPO). In summary, the combined CS–HBPL treatment successfully prolonged cucumber shelf life at room temperature, enabling new possibilities for extending cucumber shelf life.

## 1. Introduction

Cucumber (*Cucumis sativus* L.) is globally recognized as one of the top 10 vegetables and holds significant economic importance in international agriculture. Commonly referred to as green gourd, cucumber, etc., it belongs to the *Cucumis* genus within the Cucurbitaceae family [1]. Possessing a refreshing and crisp taste, cucumbers exhibit high moisture content, are abundant in essential nutrients, and can be consumed fresh or pickled. Moreover, cucumbers showcase remarkable antioxidant capacity and are rich in dietary fiber [2,3]. However, being highly perishable vegetables, cucumbers exhibit a limited shelf life of only 2–3 days when stored at room temperature. The high water content of cucumbers presents a storage challenge as they are susceptible to rotting, increased respiration, yellowing, moisture loss, and microbial proliferation even under refrigeration conditions [4,5]. In addition, postharvest cucumbers undergo vigorous physiological metabolism, rendering them susceptible to dehydration, wilting, tissue softening, head swelling, green skin fading to yellow, and decay [6]. These phenomena contribute to a rapid deterioration in nutritional quality and significantly impact both the edibility and longevity of cucumbers.

In recent years, edible films and coatings have been widely utilized for food preservation [7,8]. The semipermeable barrier provided by these coatings can effectively prolong the shelf life of fruits and vegetables by reducing mechanical damage, inhibiting microbial growth, hindering gas exchange between produce and the atmosphere, minimizing water and solute migration, and decreasing respiratory and oxidation reaction rates [9,10]. Moreover, edible coatings act as a gas barrier that creates an improved atmosphere on the surface of fruits and vegetables to delay breathing and physiological processes, thereby extending their shelf life [11]. However, a single edible film may exhibit limitations in terms of barrier properties and mechanical strength, whereas composite film materials can effectively address these issues to cater to the preservation requirements of various fruits and vegetables.

Chitosan (CS), a natural cationic polysaccharide primarily derived from crustacean shells like crab and shrimp shells [12], possesses nontoxic, biodegradable, antibacterial, and antioxidant properties. CS exhibits favorable adsorption, film-forming, and permeation characteristics due to its remarkable physical, chemical, and biological attributes. Consequently, it finds extensive applications in diverse industries such as environmental science, agriculture practices, cosmetics formulation, and food packaging for both barrier coatings or antibacterial agents [13,14,15]. Other composite films based on plant extracts have been successfully applied to improve the quality and shelf life of many fruits [16,17,18].

Hyperbranched poly-L-lysine (HBPL) is a polylysine variant characterized by a wide molecular weight distribution and a randomly branched ε-structure. The highly branched structures and abundant terminal amines of PL and PLL units in HBPL distinguish it significantly from linear polylysine (LPL) and dendritic polylysine (DPL) [19]. Chemically synthesized as an antimicrobial peptide analogue, HBPL exhibits remarkable bactericidal efficiency, excellent biocompatibility, and enhanced stability [20]. As a cationic antibacterial polymer, the antibacterial activity of HBPL relies on physical and chemical interactions with bacteria, particularly electrostatic interactions, potentially mitigating the risk of drug-resistant bacterial emergence and spread [21].

Given that L-lysine serves as the monomer for HBPL, an indispensable amino acid in human nutrition, degradation byproducts of HBPL can be deemed harmless from a biological standpoint. Due to its extensively branched structure and abundant terminal amino groups, HBPL exhibits potential applications as a carrier in gene delivery systems or drug transport vehicles [22]. Scheper et al. demonstrated that HBPL does not induce cytotoxicity or elicit adverse reactions in vivo when investigated as a drug delivery carrier [23]. Notably, the molecular structure of HBPL encompasses both ε-structural units of PL and PLL which exhibit potent antibacterial effects against Gram-positive and Gram-negative bacteria while maintaining good biological safety [19]. Hence, HBPL emerges as a promising contender for potential application as a biological preservative in the preservation of fruits and vegetables.

With the growing environmental awareness among individuals, edible coatings have emerged as a promising solution for food preservation, particularly for fruits and vegetables [24]. Research has indicated that the combination of chitosan and ε-polylysine in a composite coating was highly effective in preventing the decrease in total soluble solids (TSSs) and vitamin C (Vc) levels in citrus fruits, which significantly reduced potential damage that might occur during packaging and storage [25]. Sun et al. investigated the impact of combined treatment with chitosan and hyperbranched poly-L-lysine on the shelf life of oyster mushrooms, demonstrating its remarkable protective effect against membrane permeability damage, oxidation, and browning caused by phenolic substances [26]. However, previous studies have provided limited insights into the effects of combined treatment with chitosan and hyperbranched poly-L-lysine on cucumber preservation within this field of research. Therefore, this study aims to investigate the efficacy of a composite coating comprising chitosan and hyperbranched poly-L-lysine for preserving cucumbers.

## 2. Materials and Methods

### 2.1. Materials

Fresh cucumbers (*Cucumis sativus* L.) were purchased from Dazhang Supermarket in Luoyang City, Henan Province. The cucumbers were randomly allocated into 6 groups, with each group comprising 3 replicates containing 15 cucumbers. The selection criteria included visually intact appearance, absence of damage, and consistent size.

### 2.2. Reagent

Chitosan and thiobarbituric acid reagent were purchased from Shanghai Lanji Technology Development Co., Ltd. in Shanghai, China. Phenol, acetic acid, phenol, sodium acetate, and sodium tartrate reagents were purchased from China National Pharmaceutical Group Chemical Reagent Co., Ltd. in Beijing, China. KH_2_PO_4_, NaH_2_PO_4_, NaOH, trichloroacetic acid, glucose and vitamin C reagents were purchased from Tianjin Kemio Chemical Reagent Co., Ltd. in Tianjin, China. SOD and CAT test kits were purchased from Shanghai Shenggong Biotechnology Co., Ltd. in Shanghai, China.

### 2.3. Instruments and Equipment

Absorbance was measured using an enzyme-linked immunosorbent assay (ELISA) reader and a UV spectrophotometer, (HBS-1096B, Nanjing Detie Experimental Equipment Co., Ltd. in Nanjing, China; UV-1780, Shimadzu Corporation in Kyoto, Japan). Samples were centrifuged using a desktop freeze centrifuge. (H1850R, Hunan Xiangyi Instrument Development Co., Ltd., in Changsha, China). The permeability of the samples was measured using a desktop conductivity meter. (DDS-11C, Shanghai Precision Scientific Instrument Co., Ltd., in Shanghai, China). A constant-temperature water bath was used to bathe the samples (DK-8C, Shanghai Chaoyi Pharmaceutical Machinery Equipment Co., Ltd. in Shanghai, China).

### 2.4. Experimental Methods

#### 2.4.1. Preparation of Preservatives

Control group (CK): sterile water was used. Chitosan solution (CS): 7.5 g of chitosan was weighed and added to 500 mL of a 1% acetic acid solution; the mixture was dissolved in a constant temperature water bath at 75 °C for 30 min, followed by cooling prior to utilization. Hyperbranched poly-L-lysine solution (HBPL): 1.163 mL of hyperbranched poly-L-lysine solution with a concentration of 43% was diluted to reach a final volume of 1000 mL. Chitosan and hyperbranched poly-L-lysine complex solution (CS–HBPL): A homogeneous mixture was obtained by combining equal volumes of the CS and HBPL.

#### 2.4.2. Sample Treatment

Reapply the prepared preservative evenly onto the surface of each group of cucumbers, ensuring thorough coverage. Subsequently, transfer the cucumber groups to a cool and dry location for natural air-drying until no visible residue remained on the surface. Following, lay the cucumber groups flat on a cool and dry tabletop while maintaining room temperature at 25 °C and 45–55% relative humidity. Rotate each cucumber at least three times daily. The physical and chemical parameters were assessed at intervals of 3 days until the completion of day 18.

### 2.5. Determination Indexes and Methods

#### 2.5.1. Sensory Rating

The sensory evaluation team comprised 10 individuals, consisting of an equal distribution of 5 males and 5 females, all possessing professional expertise in experimentation [27]. The sensory evaluation table for cucumbers is shown in Table 1.

#### 2.5.2. Rot Index

Based on the deterioration extent of cucumbers, the level of decay was classified into five categories: level 0 (no rotting area), level 1 (rotting area between 0% and 5%), level 2 (rotting area ranging from 5% to 10%), level 3 (rotting area ranging from 10% to 20%), and level 4 (rotting area ranging from 20% to100%). The rot index of cucumbers can be calculated using following Formula (1) [28].

(1) $$Rot\;index=\frac{\sum \left(rot\;level\times number\;of\;cucumbers\;at\;this\;level\right)}{Total\;number\;of\;cucumbers\times highest\;level\;of\;decay}$$

#### 2.5.3. Weight Loss

Measurements were conducted by weighing. Three cucumbers were randomly selected from different experimental groups, and each cucumber was individually weighed to determine total mass changes during storage. The initial weight of each cucumber was measured using an electronic balance, followed by subsequent measurements every 3 days. Weight loss rate was calculated based on [29]:

(2) $$Weight\;loss\;rate=\frac{m_{1}-m_{2}}{m_{1}}\times 100\%$$

where m_1_ represents the initial mass of the cucumber, and m_2_ denotes the mass of the cucumber measured during each time interval.

#### 2.5.4. Hardness

Three cucumbers from distinct experimental groups were randomly selected, and their hardness was measured using a physical property analyzer without any storage period.

#### 2.5.5. Microorganism

The total number of colonies, mold, and yeast were measured according to the method described in reference [30,31].

#### 2.5.6. Soluble Protein Content

The determination was performed using the Coomassie brilliant blue G250 colorimetric method. From each group, a random selection of three cucumbers was made. A sample weighing 2.0 g was carefully ground in an ice-water bath along with 10 mL of phosphate buffer (100 mmol/L, pH 7.0). Subsequently, the mixture underwent centrifugation at a temperature of 4 °C for a duration of 25 min at a speed of 8000 r/min. Following this step, 1 mL of the resulting supernatant was collected. Subsequently, 5 mL of Coomassie brilliant blue G250 reagent was added to the supernatant, mixed well, and allowed to stand for 2 min before measuring the absorbance at 595 nm. The protein concentration was determined by utilizing a standard curve derived from bovine serum albumin.

#### 2.5.7. Vitamin C Content

The determination of vitamin C content in cucumbers was conducted following the titration method described in reference [32], using 2,6-dichloro indophenol as the indicator. Three cucumbers were randomly selected from each group and weighed at 10.0 g per sample. The samples were then homogenized with a 20.0 g/L oxalic acid solution to obtain a final volume of 100 mL. After filtration, 10 mL of the filtrate was transferred into a conical flask for titration with a calibrated solution of 2,6-dichloro indophenol. The endpoint was reached when the solution turned slightly red and remained stable for at least 15 s. A blank experiment was performed using 10 mL of the oxalic acid solution (20.0 g/L). The vitamin C content was calculated according to the following formula:

(3) $$\mathrm{Vitamin}\;C\;\mathrm{content}=\frac{\left(v-v_{0}\right)\times T\times A}{M}\times 100\%$$

In the formula, V denotes the amount of 2,6-dichloro indophenol solution utilized in titrating the sample solution (mL); V_0_ refers to the quantity of 2,6-dichloro indophenol solution consumed during titration of the blank (mL); T is the milligram equivalent of ascorbic acid per milliliter of 2,6-dichloro indophenol solution (mg/mL); A is the dilution factor; and M is the mass of the sample (g).

#### 2.5.8. Reducing Sugar Content

The degree of sugar reduction in cucumbers was assessed utilizing the colorimetric method with 3,5-dinitrosalicylic acid. Cucumber liquid that had been filtered underwent appropriate dilution and was subsequently combined with 1 mL of a diluent solution and 0.75 mL of DNS within a test tube. The mixture was heated in a boiling water bath for 5 min, followed by cooling to room temperature. Distilled water was added to reach a final volume of 20 mL, and the solution was thoroughly mixed by inversion before measuring the absorbance at a wavelength of 540 nm.

#### 2.5.9. Total Phenolic Content

Quantification of the overall phenolic levels was conducted through utilization of the Folin phenol method [33]. Initially, 5 g of cucumber was weighed and mixed with a small amount of precooled 1% HCl methanol solution. The mixture was homogenized in an ice bath and transferred to a 20 mL graduated test tube. Subsequently, the volume was brought to the designated level using a 1% HCl methanol solution and subjected to ultrasonic extraction for a duration of 30 min. Following centrifugation at 10,000 r/min for 30 min at a temperature of 4 °C, the resulting liquid was gathered. Then, 0.5 mL of supernatant was taken into a 10 mL colorimetric tube and mixed with 1 mL of phenol reagent. After standing in darkness for 5 min, it was further treated with the addition of 3 mL of a 20% Na_2_CO_3_ solution and water up to a final volume of 10 mL. The mixture obtained was allowed to stand at ambient temperature for a duration of 2 h prior to assessing the absorbance at a wavelength of 765 nm against the standard curve that had been established using gallic acid.

#### 2.5.10. Malondialdehyde (MDA)

The determination was performed using the thiobarbituric acid method [34].

Three cucumbers were randomly selected from each group, and 1.0 g of each sample was finely crushed in a solution of 5 mL TCA with a concentration of 10%. The mixture was then centrifuged at 10,000 r/min for 10 min, and 2 mL of the resulting supernatant was taken. To the supernatant, a solution of thiobarbituric acid at 0.6% concentration was added in equal volume with distilled water and mixed thoroughly. The mixture was boiled in water for 20 min, cooled naturally, and then centrifuged at 8000 r/min for another 10 min. Finally, the absorbance of the supernatant at wavelengths of 450 nm, 532 nm, and 600 nm was measured.

(4) $$MDA\;content=\frac{\left[6.45\times \left(A_{532}-A_{600}\right)-0.56A_{450}\right]\times V_{1}\times V_{3}}{V_{2}\times M}$$

In the formula, A_450_, A_532_, and A_600_ represent absorbance values; V_1_ is the total volume of the solution obtained after measurement; V_2_ is the volume of liquid extracted during measurement; V_3_ represents the total volume of extraction liquid; and M is the mass of the sample.

#### 2.5.11. Electrolyte Leakage Rate

Three cucumbers were randomly selected from each group, and cucumber flesh slices with a thickness of approximately 0.5 cm were obtained. The surface electrolyte of the cucumber was rinsed with 40 mL of deionized water, followed by drying using filter paper before transferring it to a 15 mL beaker containing deionized water. The mixture was stirred at 25 °C for 30 min using a magnetic stirrer to measure the initial conductivity (C_0_). Conductivity measurements (C_1_) were taken after 10 min, and final conductivity (C_2_) was measured after boiling the sample for 10 min and cooling it to room temperature.

(5) $$Electrolyte\;leakage\;rate(\%)=\frac{C_{1}-C_{0}}{C_{2}-C_{0}}$$

#### 2.5.12. Enzymatic Activity

Catalase (CAT), superoxide dismutase (SOD), and polyphenol oxidase (PPO) assay kits were utilized to assess enzyme activity in cucumber during various storage stages.

### 2.6. Statistical Analysis

The mean ± SD (standard deviation) was used to represent the experimental results. Statistical analysis was conducted using SPSS 18.0, and a significance level of *p* < 0.05 was considered statistically significant.

## 3. Results and Discussion

### 3.1. Effects of CS and HBPL Treatments on Sensory Scores of Cucumber during Storage

**Figure 1:**
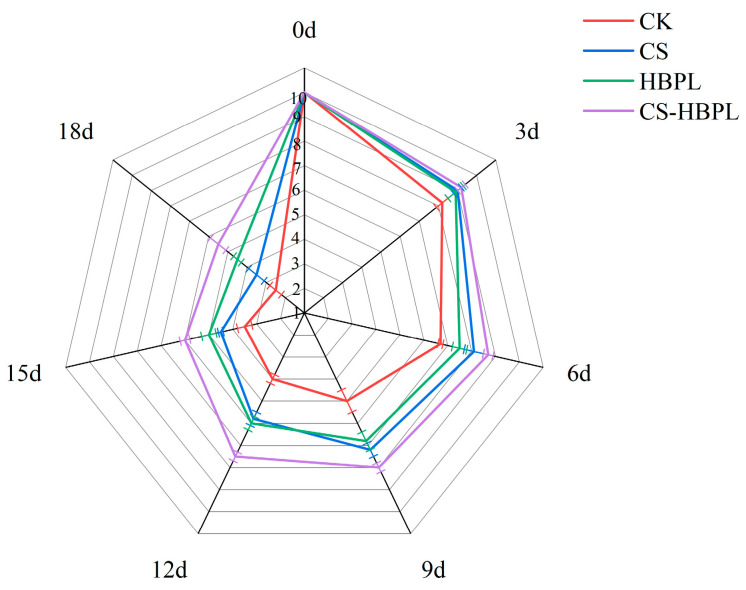
Effects of CS and HBPL treatments on sensory scores of cucumber during storage.

The freshness duration of fruits and vegetables pertains to the period they can be stored without compromising their sensory quality or exceeding the safety limit for microbial count [35]. Sensory quality serves as a crucial indicator for consumers to assess cucumber quality. The sensory score of cucumbers decreased with prolonged storage time (Figure 1). By day 3 of storage, there was no significant decrease in sensory evaluation for the CS, HBPL, or CS–HBPL group: they maintained the expected color, odor, and hardness characteristics of cucumbers. In comparison to the other three groups, the CK group exhibited slightly darker cucumber color, lighter aroma, and decreased hardness. The cucumbers from the CK group exhibited surface yellowing, a pronounced sour taste, and noticeable softening throughout the body after 12 days of storage. Cucumbers from the CS and HBPL groups showed slight yellowing on their surface with only a mild sour taste and minimal softening observed in their bodies, while cucumbers from the CS–HBPL group had a slightly yellowish surface without any sour taste, but demonstrated fuller firmness. On the 18th day of storage, extensive yellowing was observed in the melon bodies of CK, CS, and HBPL groups, accompanied by pronounced sour taste and significant softening, with the CK group most severe. The CS–HBPL group showed severe overall softness but no apparent sour taste, while the yellow and green areas on the cucumber surface remained unchanged. Compared to the CK group, all groups receiving treatment demonstrated the ability to prevent alterations in color, odor, and hardness of cucumber. Notably, the CS–HBPL group exhibited the most pronounced inhibitory effect.

### 3.2. Effects of CS and HBPL Treatments on the Rot Index, Weight Loss, and Hardness of Cucumber during Storage

**Figure 2:**
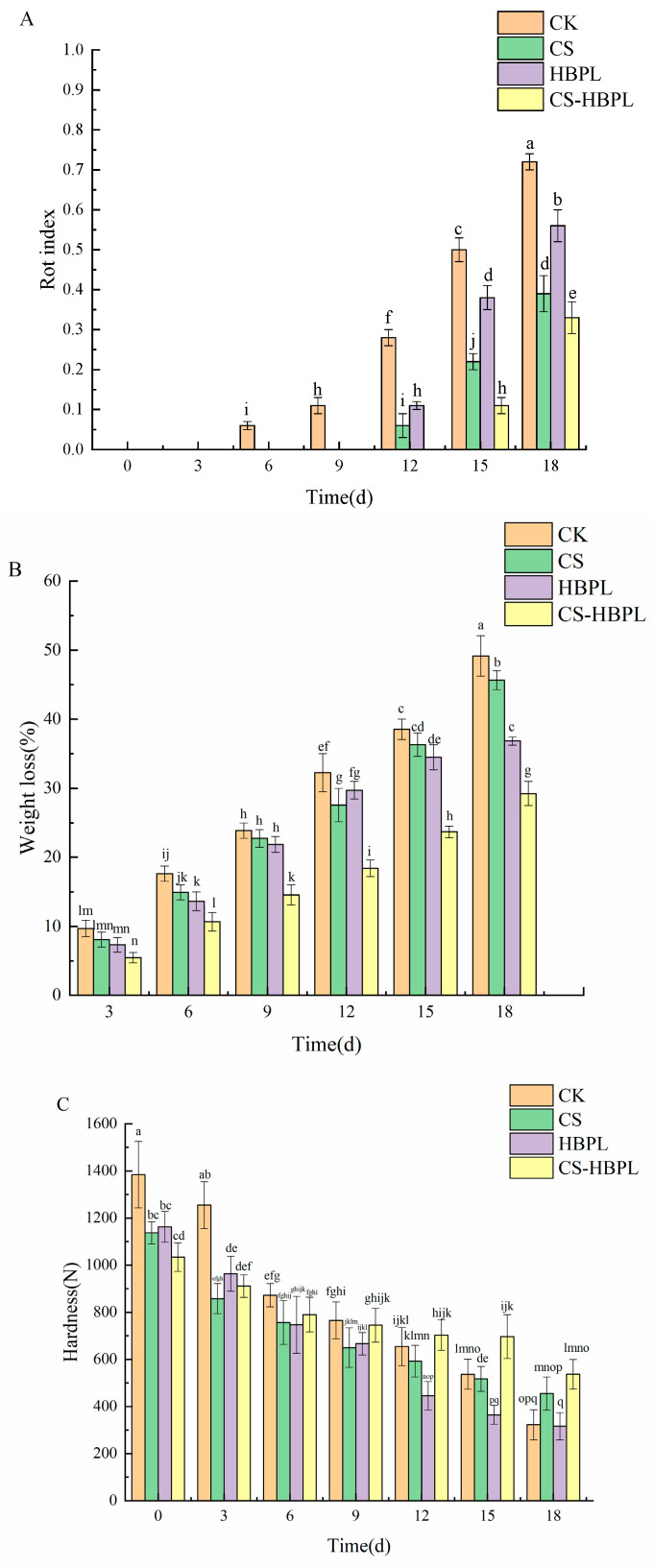
Effects of CS and HBPL treatments on the rot index, weight loss, and hardness of cucumber during storage. (**A**) Rot index; (**B**) weight loss; (**C**) hardness. Standard deviations are represented by vertical bars. Values labeled with different letters indicate significant variations (*p* < 0.05).

The deterioration of fresh fruits and vegetables directly influences consumer purchasing inclination, while the rot index serves as a crucial indicator for assessing cucumber quality. The rot index of cucumbers increased with prolonged storage time (Figure 2A). No signs of decay were observed in any of the four groups on day 3 of storage. On the days 6 and 9, cucumbers began to exhibit rotting symptoms in the CK group, whereas no decay was observed in the other three groups. On day 12 of storage, cucumbers in both the CS and HBPL groups started rotting; however, the rot index exhibited a significantly lower value compared to that observed in the CK group (*p* < 0.05). From the 15th day of storage, cucumbers belonging to the CS–HBPL group exhibited a noticeably higher level of deterioration compared to the other three groups (*p* < 0.05). On day 18 of storage, the rot indexes of the CK, CS, HBPL, and CS–HBPL groups were 0.72, 0.39, 0.56, and 0.33, respectively. Compared to the CK group, the inhibitory effect against cucumber decay was significant in all three treatment groups (*p* < 0.05), and the CS–HBPL group exhibited the most pronounced inhibitory effect.

Fruits and vegetables are prone to experiencing weight reduction while being stored, which can affect their visual appeal, texture, nutritional value, and taste. The rate of weight loss in cucumbers escalated as the duration of storage was prolonged (Figure 2B). Throughout the entire storage period, the weight loss rates in the three treatment groups were lower compared to the control group. On day 18 of storage, the weight loss rates for the CK, CS, HBPL, and CS–HBPL groups were 49.18%, 45.62%, 36.84%, and 29.25%, respectively. Notably, a significantly lower weight loss rate was observed in the CS–HBPL group than the CS, HBPL, and CK groups when examining cucumbers (*p* < 0.05).

The hardness of cucumbers decreased with the extension of storage time (Figure 2C). On day 9 of storage, compared to day 0, the hardness reduction in CK, CS, HBPL and CS–HBPL groups was 44.70%, 42.86%, 42.68% and 27.95%, respectively. Notably, the hardness reduction in the CS–HBPL group was found to be significantly less than the CK, CS, and HBPL groups (*p* < 0.05). The hardness of cucumbers in the CK, CS, HBPL, and CS–HBPL groups decreased by 76.67%, 60.00%, 72.80%, and 48.09%, respectively, after storage for 18 days compared to day 0. Significantly less reduction in cucumber hardness was observed in the CS–HBPL group than the CK, CS, and HBPL groups (*p* < 0.05). These findings demonstrated that treatment with CS–HBPL effectively inhibited cucumber rot.

Fan et al. employed modified air packaging and chitosan carbon dot coating to preserve freshly cut cucumbers, effectively reducing the rate of weight loss and maintaining their firmness [36]. Olawuyi et al. employed a blend of 2% chitosan covering along with modified atmosphere packaging to handle freshly sliced cucumbers, resulting in reduced quality deterioration and respiration rate [37]. These findings aligned with the outcomes observed in this study.

### 3.3. Effects of CS and HBPL Treatments on Microorganisms during Cucumber Storage

**Figure 3:**
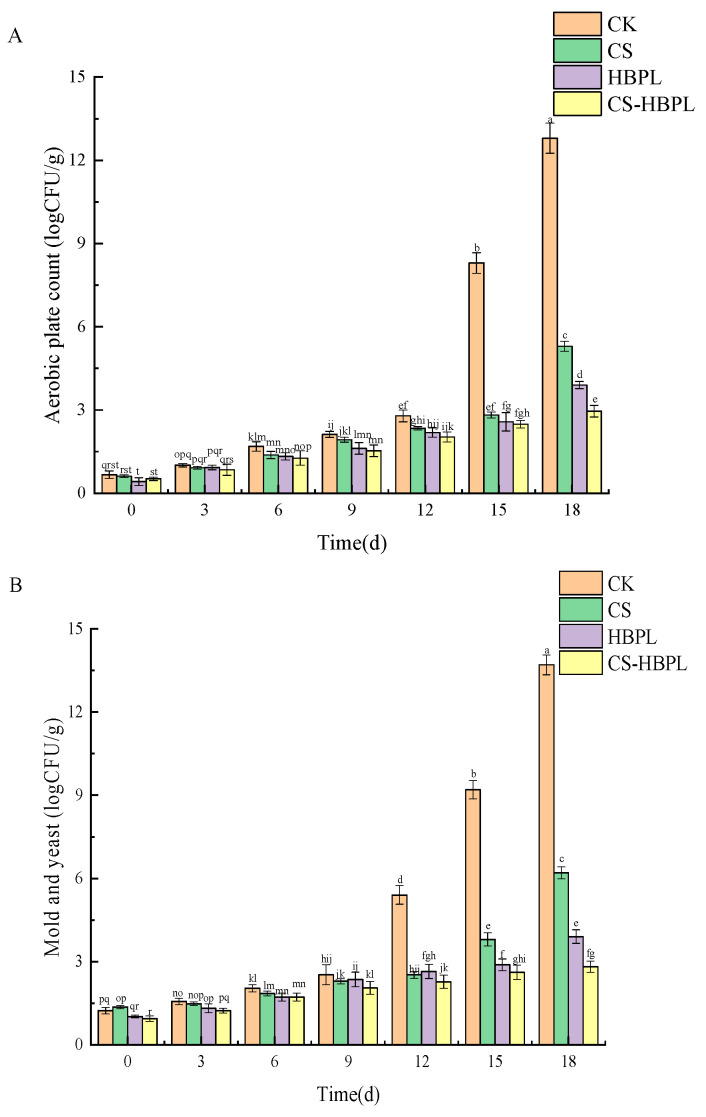
Effects of CS and HBPL treatments on microorganisms during cucumber storage. (**A**) Aerobic plate count; (**B**) mold and yeast. Standard deviations are represented by vertical bars. Values labeled with different letters indicate significant variations (*p* < 0.05).

Fresh fruits and vegetables are highly susceptible to microbial contamination, which can easily lead to spoilage and significantly impact their commercial value. The quantity of bacterial colonies, mold, and yeast present in cucumbers exhibited an upward trend as the duration of storage increased (Figure 3). After 18 days of storage, total colonies in the CK, CS, HBPL, and CS–HBPL groups were 12.8 logCFU/g, 5.3 logCFU/g, 3.9 logCFU/g, and 2.96 logCFU/g, respectively, and mold and yeast in the CK, CS, HBPL, and CS–HBPL groups were 13.7 logCFU/g, 6.2 logCFU/g, 3.9 logCFU/g, and 2.81 logCFU/g, respectively. The number of colonies, as well as mold and yeast in the CS, HBPL, and CS–HBPL groups, were significantly lower compared to those in the CK group (*p* < 0.05), which suggested that treatment with CS and HBPL effectively inhibited the increase of total colony count, mold, and yeast during cucumber storage. Furthermore, the number of colonies, mold and yeast in CS–HBPL group were significantly lower than those in CS and HBPL group (*p* < 0.05), indicating that CS–HBPL treatment had the most pronounced inhibitory effect on colony count, mold, and yeast. This phenomenon might be attributed to the coating’s ability to create an enhanced environment that effectively retarded the growth rate of cucumber surface spoilage and pathogenic microorganisms [11].

### 3.4. Effects of CS and HBPL Treatments on Soluble Protein, Vc Reducing Sugar, and Total Phenols during Cucumber Storage

**Figure 4:**
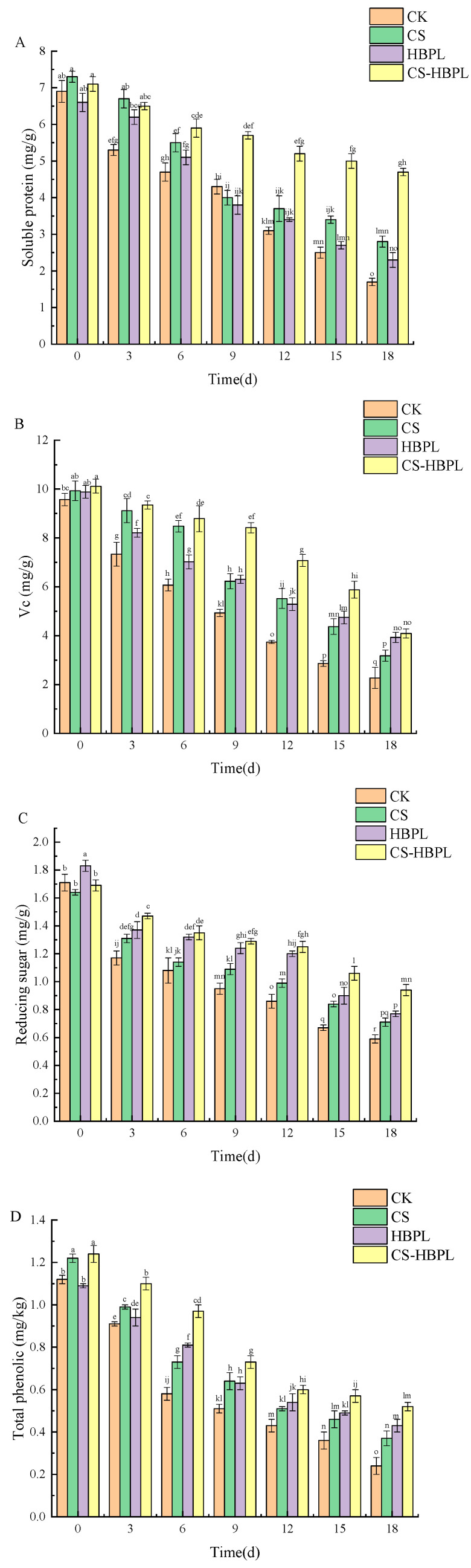
Effects of CS and HBPL treatments on soluble protein, Vc, reducing sugar, and total phenols during cucumber storage. (**A**) Soluble protein; (**B**) Vc; (**C**) reducing sugar; (**D**) total phenolic content. Standard deviations are represented by vertical bars. Values labeled with different letters indicate significant variations (*p* < 0.05).

Soluble proteins play a crucial role in osmotic regulation, and their decreased content can disrupt cellular osmotic pressure balance. The soluble protein content of cucumber gradually declined during storage (Figure 4A). Starting from day 3, there was an observable decline in the level of soluble protein content, and the experimental groups demonstrated a significantly greater amount of soluble protein content when compared to the control group (*p* < 0.05). On day 18 of storage, the cucumbers of CK, CS, HBPL, and CS–HBPL groups had soluble protein of 1.7 mg/g, 2.8 mg/g, 2.3 mg/g, and 4.7 mg/g, respectively. The soluble protein content in the treatment groups exhibited a statistically significant increase compared to that of the control group (*p* < 0.05), and the CS–HBPL group exhibited notably higher levels compared to CS, HBPL groups (*p* < 0.05). On the 18th day of storage, there was a significant reduction in soluble protein content observed in the CK, CS, HBPL, and CS–HBPL groups. The decrease percentages were 75.36%, 61.64%, 65.15%, and 33.80%, respectively, compared to the initial storage period. It could be observed that application of CS–HBPL effectively mitigated soluble protein loss in cucumbers. A possible reason was that the inhibition of respiration rate and the delay in protein and carbohydrate breakdown by CS–HBPL coating contributed to the prevention of postharvest cucumber aging.

Vitamin C is a vital nutrient for the human body, and its content serves as one of the evaluation indicators for assessing the nutritional quality and storage efficacy of fruits and vegetables. However, due to its inherent instability and susceptibility to oxidation, vitamin C can degrade under various factors such as pH, water activity, and enzymes [38]. The Vc content in cucumbers gradually decreased with prolonged storage time (Figure 4B). On day 9 of storage, each treatment group exhibited significantly higher Vc content compared to the CK group (*p* < 0.05). Notably, the CS–HBPL group exhibited substantially higher levels than both the CS and HBPL groups (*p* < 0.05). By day 18 of storage, the Vc content in the CK, CS, HBPL, and CS–HBPL groups was 2.27 mg/g, 3.18 mg/g, 3.93 mg/g, and 4.09 mg/g, respectively. In comparison to initial measurements, this represented a decrease by 76.28%, 67.98%, 60.22%, and 59.58%, respectively, across all groups examined. The Vc content of the CS–HBPL group was found to be significantly higher than the other groups (*p* < 0.05). These findings indicated that CS–HBPL treatment could effectively inhibit reduction of Vc in cucumber. The findings of this study were consistent with those reported by Huang et al. [8], who observed higher levels of ascorbic acid in *Lentinus edodes* coated with a composite edible coating made from chitosan and guar gum.

The content of reducing sugars in cucumbers gradually decreased with the prolongation of storage time (Figure 4C). On the 9th day of storage, the levels of reducing sugars were significantly elevated in the CS, HBPL, and CS–HBPL groups compared to the CK group (*p* < 0.05). By day 18 of storage, the content of reducing sugars in the CK, CS, HBPL, and CS–HBPL groups was 0.59 mg/g, 0.71 mg/g, 0.77 mg/g, and 0.94 mg/g, respectively. The CK group exhibited significantly lower reducing sugar content compared to all treatment groups (*p* < 0.05). Compared to the initial day, the CK, CS, HBPL, and CS–HBPL group showed reductions in sugar content by 65.50%, 56.71%, 57.92%, and 44.38%, respectively. This indicated that the CS–HBPL treatment could effectively inhibit the reduction of reducing sugar in cucumbers. Similar results were obtained by Fan et al. [39], who found that carbon dots with chitosan coating could significantly reduce the loss of ascorbic acid in freshly cut cucumber.

The changes in phenolic substances have an impact on the browning process of fruits and vegetables. A decrease in phenolic substances indicates a decline in the freshness of fruits and vegetables [40]. The total phenolic content in cucumbers gradually decreased with prolonged storage time (Figure 4D). On the 6th day of storage, the treatment groups demonstrated notably elevated levels of total phenolic content in comparison to the CK group (*p* < 0.05), with CS–HBPL showing particularly significant. By day 18 of storage, the content of total phenolic in the CK, CS, HBPL, and CS–HBPL groups was 0.24 mg/kg, 0.37 mg/kg, 0.43 mg/kg, and 0.52 mg/kg, representing a reduction by 78.57%, 69.67%, 60.55%, and 58.06% from the initial storage date, respectively. The CS–HBPL group demonstrated significantly higher total phenolic content than the other groups (*p* < 0.05), indicating that the CS–HBPL treatment could effectively inhibit the reduction of total phenolic in cucumbers. The study conducted by Yuan et al. [41] yielded similar findings, demonstrating that the application of chitosan coating significantly mitigated the loss of total phenol in mini-cucumber.

### 3.5. The Effects of CS and HBPL Treatments on the Permeability of Malondialdehyde and Relative Electrolyte during Cucumber Storage

**Figure 5:**
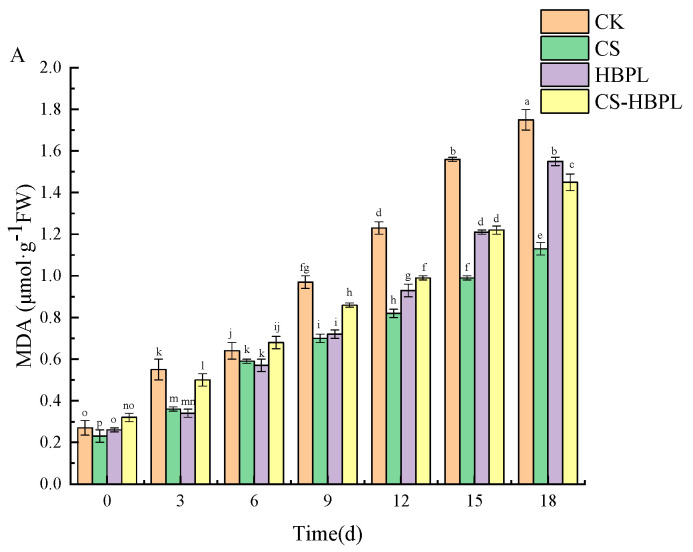

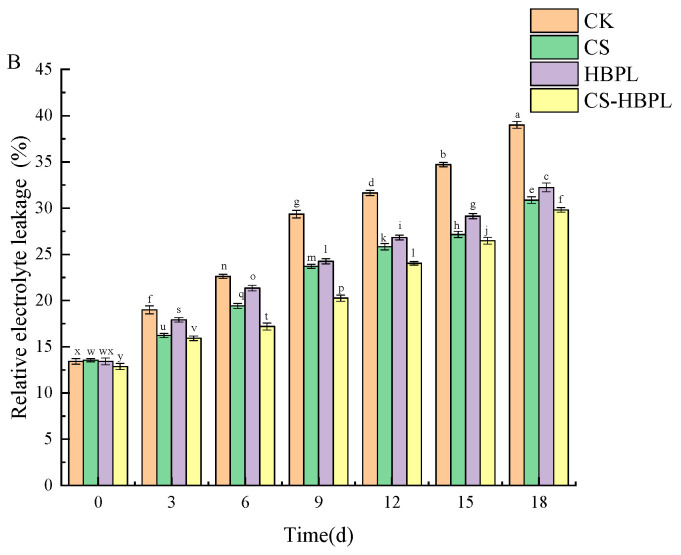
The effects of CS and HBPL treatments on the permeability of MDA and relative electrolyte leakage during cucumber storage. (**A**) MDA; (**B**) relative electrolyte leakage. Standard deviations are represented by vertical bars. Values labeled with different letters indicate significant variations (*p* < 0.05).

The primary outcome of lipid peroxidation in the membranes of plant cells is MDA. When fruit and vegetable tissues age or are damaged, the membrane system undergoes lipid peroxidation reactions, leading to the production of MDA, and the changes in MDA content can serve as an indicator of cell membrane damage [42]. The MDA content in cucumbers gradually increased with prolonged storage time (Figure 5A). On day 3 of storage, the MDA content in cucumbers from the control group exhibited a significantly higher level compared to other treatment groups (*p* < 0.05). On day 18 of storage, the MDA content in cucumbers from CK, CS, HBPL, and CS–HBPL groups were 1.75 μmol·g^−1^FW, 1.13 μmol·g^−1^FW, 1.55 μmol·g^−1^FW, and 1.45 μmol·g^−1^FW, respectively, which represented an increase of 84.57%, 79.64%, 83.23%, and 77.93% compared to the initial storage day. The CS–HBPL treatment demonstrated a significant reduction in MDA content in cucumbers compared to the CK group (*p* < 0.05), suggesting its effective inhibition of MDA accumulation.

In the course of fruit and vegetable storage, enhanced permeability is observed as a result of cellular membrane impairment, resulting in electrolyte leakage within the cells and subsequently elevated relative conductivity of fruit extracts. Therefore, measuring cell membrane permeability using a conductivity meter can assess the relative conductivity of cucumbers during storage. The cucumbers’ electrolyte leakage rate exhibited a gradual increase as the storage time prolonged (Figure 5B). On day 3 of storage, the treatment group exhibited significantly lower relative electrolyte leakage rate than the CK group (*p* < 0.05). On day 18 of storage, the relative electrolyte leakage rate in the CK, CS, HBPL, and CS–HBPL groups was 39.01%, 30.88%, 32.24%, and 29.82%, respectively. Notably, the CS–HBPL group demonstrated a significantly lower relative electrolyte leakage rate than the other groups (*p* < 0.05). These findings suggested that CS–HBPL treatment could effectively mitigate the relative electrolyte leakage increase in cucumber. Huang et al. [8] obtained similar findings, demonstrating that the application of chitosan and guar gum composite edible coating effectively inhibited the elevation of MDA levels and relative electrolyte leakage in *Lentinus edodes*.

### 3.6. Effects of CS and HBPL Treatments on Enzyme Activity during Cucumber Storage

**Figure 6:**
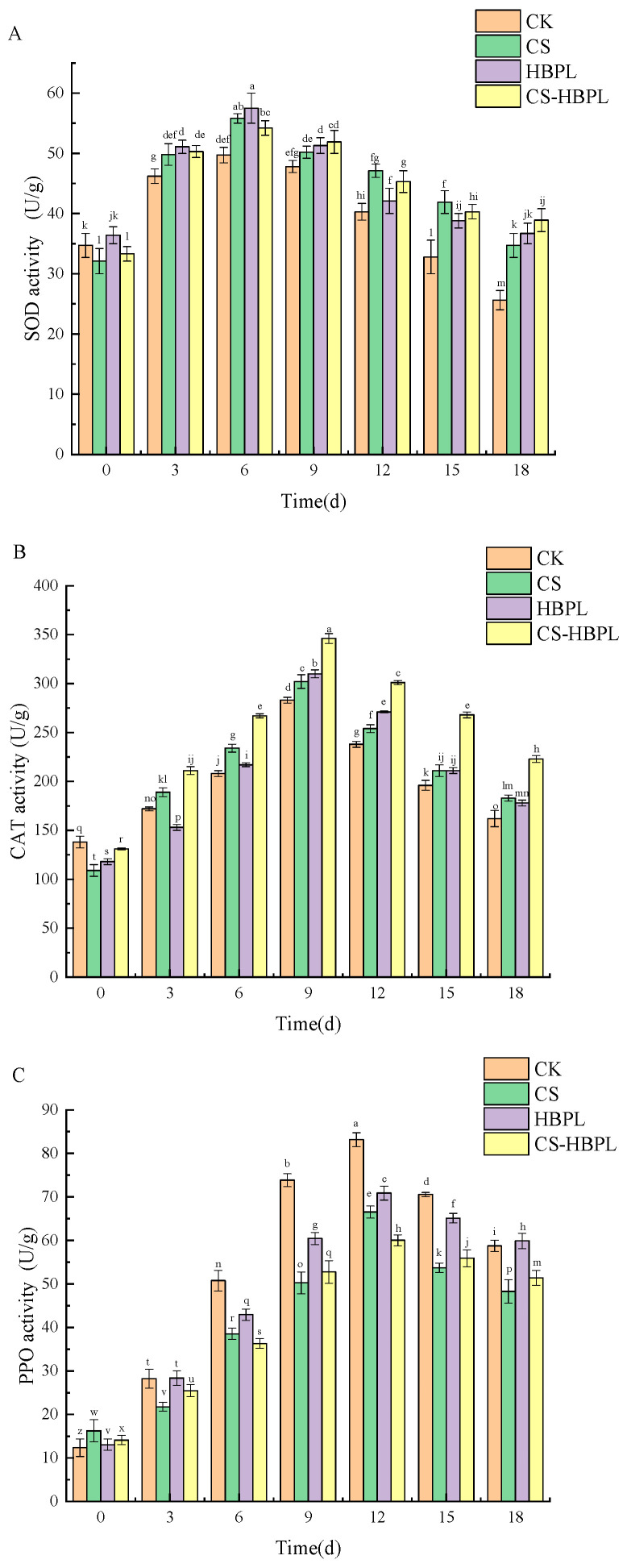
Effects of CS and HBPL treatments on enzyme activity during cucumber storage. (**A**) SOD activity; (**B**) CAT activity; (**C**) PPO activity. Standard deviations are represented by vertical bars. Values labeled with different letters indicate significant variations (*p* < 0.05).

SOD plays a crucial role in maintaining the balance of reactive oxygen species metabolism by clearing superoxide anions and generating H_2_O_2_ and O_2_, thereby exerting a protective effect on cellular integrity. The SOD activity of cucumber exhibited an initial increase followed by a subsequent decrease during storage (Figure 6A). On day 6, the SOD activity reached its peak value in all groups, with values of 49.7 U/g in the CK group, 55.8 U/g in the CS group, 57.5 U/g in the HBPL group, and 54.2 U/g in the CS–HBPL group. The SOD activity in the CK group was found to be significantly lower in comparison to that of the treatment groups (*p* < 0.05). Subsequently, there was a gradual decline in SOD activity across all groups after 6 days of storage. By day 18, SOD activity had decreased to levels of 25.6 U/g in CK group, 34.7 U/g in CS group, 36.7 U/g in HBPL group, and 38.9 U/g in CS–HBPL group, respectively; the CS–HBPL group maintained significantly higher SOD activity compared to other groups (*p* < 0.05). The findings indicated that treatment with CS–HBPL could effectively delay the reduction of SOD activity and mitigate cell membrane damage during cucumber storage.

The CAT enzyme effectively eliminates H_2_O_2_ generated by plant metabolism, decomposing it into nontoxic H_2_O and O_2_. The CAT activity of cucumber exhibited an initial increase followed by a subsequent decrease during the storage period (Figure 6B). On day 9 of storage, the CAT activities of cucumber in the CK, CS, HBPL, and CS–HBPL groups reached their highest values at 283 U/g, 302 U/g, 310 U/g, and 346 U/g respectively. CAT activities in the treated groups were significantly higher than those in the CK group (*p* < 0.05), and the CS–HBPL group showed significantly higher CAT activity compared to both the CS and HBPL groups (*p* < 0.05). After day 9 of storage, all groups showed a decreasing trend in CAT activity of cucumber. By day 18 of storage, CAT activities in the CK, CS, HBPL, and CS–HBPL groups had decreased to 162 U/g, 183 U/g, 178 U/g, and 223 U/g, respectively. CAT activity in the CS–HBPL group was significantly higher than that in the other groups (*p* < 0.05). These results indicated that treatment with CS–HBPL could effectively inhibit decreases in CAT activity of cucumber. The elevation in CAT activity could effectively eliminate hydrogen peroxide, mitigate the oxidative damage caused by free radicals, and suppress the enzymatic browning process of cucumbers.

The PPO activity of cucumber showed an initial increase followed by a decrease with prolonged storage time (Figure 6C). On day 12 of storage, the PPO activity in cucumbers from the CK, CS, HBPL, and CS–HBPL groups reached peaks of 83.16 U/g, 66.54 U/g, 70.86 U/g, and 60.01 U/g, respectively. Notably, the CS–HBPL-treated cucumbers exhibited significantly lower PPO activity than the other groups (*p* < 0.05). After 12 days of storage, PPO activity in cucumbers started to decline for all groups. By day 18, cucumbers treated with CS–HBPL had significantly lower PPO activity than the CK group (*p* < 0.05). These results indicated that CS–HBPL treatment could effectively inhibit the elevation of PPO activity in cucumbers. The membrane system of cucumbers is damaged as storage time increases after harvesting, leading to an increase in PPO activity. However, during the later stages of storage, the oxygen consumption of the fruit decreased and PPO activity started to decline [43].

## 4. Conclusions

The present study utilized fresh cucumbers as experimental materials to assess the effects of CS and HBPL treatments on sensory quality, rot index, weight loss, hardness, microbial determination, vitamin C, total phenolic, reducing sugar, MDA levels, relative electrolyte permeability, CAT, SOD, and PPO activity during cucumber storage. Experimental findings demonstrated that compared to the CK group and individual CS or HBPL treatment group, cucumbers treated with a combination of CS–HBPL exhibited significantly higher sensory evaluation scores. On day 18 of storage, the cucumbers in the CK group showed significant decay and softening, while no decay or noticeable sour taste was observed in the CS–HBPL group despite decreased hardness. Furthermore, the CS–HBPL treatment effectively delayed cucumber decay progression and weight loss rate increase while inhibiting both cucumber surface microorganism growth and decrease in cucumber hardness when compared to the CK group. Additionally, it significantly reduced soluble protein loss, Vc degradation, and reduced sugar depletion and total phenol reduction in cucumbers. Furthermore, the combined treatment significantly suppressed MDA accumulation and the increase in relative electrolyte leakage. In addition, it markedly enhanced SOD and CAT activities while inhibiting PPO activity. Comprehensively, the CS–HBPL treatment effectively preserved cucumber quality characteristics, delayed nutrient loss, sustained appearance integrity, inhibited phenolic substance oxidation, elevated cucumber hardness decline rate, and improved commercial value. The combined treatment of CS–HBPL not only effectively suppresses the surface microbial growth and respiratory metabolism of cucumber, thereby prolonging its shelf life through internal and external interactions, but also demonstrates excellent biosafety. This innovative approach provides valuable technical information for large-scale storage and transportation of cucumbers, while holding potential for application in the preservation of freshness in various fruits and vegetables.

## Figures and Tables

**Table 1:** Sensory evaluation standards for *Cucumis sativus* L.

Score	Color and Luster	Smell	Hardness
9~10	Bright, glossy, and dark green in color.	Has a strong fresh cucumber fragrance.	High hardness, full body, no softening, no rotten heads.
7~8	Relatively bright, slightly dark in color, appearing green.	There is a cucumber fragrance, but it is slightly light.	The body is harder, fuller, slightly softened, and has no rotten heads.
5~6	The color is dim, appearing light green with a slight yellowing.	Cucumber has a lighter fragrance and a slight sour taste.	The body begins to become soft, not full, with significant water loss, and the tail slightly collapses.
3~4	Yellow is obvious, with yellow area greater than green area.	No cucumber fragrance, with a strong sour taste.	The body has noticeably softened, lost more water, has severe wrinkles, and the tail has collapsed significantly, resulting in a large head phenomenon.
1~2	The overall color is yellow, with a slight hint of green.	There is a clear cucumber sour taste.	Most of the body softens, with severe dehydration, wilting of the body, severe collapse of the tail, and significant swelling of the head.

## Data Availability

The original contributions presented in the study are included in the article, further inquiries can be directed to the corresponding author.

## References

[1] Che G., Zhang X.L. (2019). Molecular basis of cucumber fruit domestication. Curr. Opin. Plant Biol..

[2] İbrahim K., Serhat U. (2019). Improving Postharvest Storage Quality of Cucumber Fruit by Modified Atmosphere Packaging and Biomaterials. HortScience.

[3] Shi J., Wang J.F., Li R., Li D.B., Xu F.F., Sun Q.J., Zhao B., Mao A.J., Guo Y.D. (2015). Expression patterns of genes encoding plasma membrane aquaporins during fruit development in cucumber (*Cucumis sativus* L.). Plant Physiol. Biochem..

[4] María L., David Q.G., Ricardo M.G.R., María A.C.A., Gerardo G.L., Zaida U.M. (2021). Effects of UV-C and Edible Nano-Coating as a Combined Strategy to Preserve Fresh-Cut Cucumber. Polymers.

[5] Olawuyi F.I., Lee W. (2019). Influence of chitosan coating and packaging materials on the quality characteristics of fresh-cut cucumber. Korean J. Food Preserv..

[6] Bao Y.Q., Chen Y.P., Han Y.Y. (2023). Postharvest physiological changes and storage techniques of cucumber: Research progress. Chin. Agric. Sci. Bull..

[7] Liu Q., Cui X., Song Z.B., Kong W.W., Kang Y.C., Kong W.L., Ng T.B. (2021). Coating shiitake mushrooms (*Lentinus edodes*) with a polysaccharide from oudemansiella radicata improves product quality and flavor during postharvest storage. Food Chem..

[8] Huang Q.H., Qian X.C., Jiang T.J., Zheng X.L. (2019). Effect of chitosan and guar gum based composite edible coating on quality of mushroom (*Lentinus edodes*) during postharvest storage. Sci. Hortic..

[9] Ali S., Khan A.S., Nawaz A., Anjum M.A., Naz S., Ejaz S., Hussain S. (2019). Aloe vera gel coating delays postharvest browning and maintains quality of harvested litchi fruit. Postharvest Biol. Technol..

[10] Liu Q., Kong W., Hu S., Kang Y., Zhang Y., Ng T.B. (2020). Effects of *Oudemansiella radicata* polysaccharide on postharvest quality of oyster mushroom (*Pleurotus ostreatus*) and its antifungal activity against *Penicillium digitatum*. Postharvest Biol. Technol..

[11] Graü M.A.R., Tapia M.S., Belloso O.M. (2008). Using polysaccharide-based edible coatings to maintain quality of fresh-cut Fuji apples. LWT-Food Sci. Technol..

[12] Tan X.Y., Liu Y., Wu X.X., Geng M.J., Teng F. (2024). Layer-by-layer self-assembled liposomes prepared using sodium alginate and chitosan: Insights into vesicle characteristics and physicochemical stability. Food Hydrocoll..

[13] Ma Z.X., Garrido-Maestu A., Jeong C.K. (2017). Application, mode of action, and in vivo activity of chitosan and its micro- and nanoparticles as antimicrobial agents: A review. Carbohydr. Polym..

[14] Chi K., Catchmark M.J. (2018). Improved eco-friendly barrier materials based on crystalline nanocellulose/chitosan/carboxymethyl cellulose polyelectrolyte complexes. Food Hydrocoll..

[15] Oyervides-Muñoz E., Pollet E., Ulrich G., Sosa-Santillán G.G., Avérous L. (2017). Original method for synthesis of chitosan-based antimicrobial agent by quaternary ammonium grafting. Carbohydr. Polym..

[16] Zhang W.L., Li X.X., Jiang W.B. (2020). Development of antioxidant chitosan film with banana peels extract and its application as coating in maintaining the storage quality of apple. Int. J. Biol. Macromol..

[17] González-Saucedo A., Barrera-Necha L.L., Ventura-Aguilar I.R., Correa-Pacheco Z.N., Bautista-Baños S., Hernández-López M. (2019). Extension of the postharvest quality of bell pepper by applying nanostructured coatings of chitosan with *Byrsonima crassifolia* extract (L.) Kunth. Postharvest Biol. Technol..

[18] Adilah A.N., Noranizan M.A., Jamilah B., Hanani Z.N. (2020). Development of polyethylene films coated with gelatin and mango peel extract and the effect on the quality of margarine. Food Packag. Shelf.

[19] Yang Z.J., Xi Y., Bai J., Jiang Z.W., Wang S.Q., Zhang H.L., Dai W., Chen C.Z., Gou Z.R., Yang G.L. (2020). Covalent grafting of hyperbranched poly-L-lysine on Ti-based implants achieves dual functions of antibacteria and promoted osteointegration in vivo. Biomaterials.

[20] Chen Q., Xu Y.J., Feng J.Y., Lv X.S., Fu X.H., Yuan S.S., Li Z.B. (2023). Hyperbranched Poly-L-Lysine Based Water-Insoluble Complexes as Antibacterial Agents with Efficient Antibacterial Activity And Cytocompatibility. Macromol. Biosci..

[21] Lu H.D., Tu C.X., Zhou T., Zhang W.Y., Zhan Y.B., Ding J., Wu X.Y., Yang Z.J., Cao W.B., Deng L.W. (2022). A ROS-scavenging hydrogel loaded with bacterial quorum sensing inhibitor hyperbranched poly-L-lysine promotes the wound scar-free healing of infected skin in vivo. Asia-Pac. J. Chem. Eng..

[22] Peng Q., Zhu J.J., Yu Y.S., Hoffman L., Yang X.K. (2015). Hyperbranched lysine-arginine copolymer for gene delivery. J. Biomat. Sci. Polym. E.

[23] Verena S., Melanie W., Markus S., Zuzana K., Thomas P., Harm-Anton K., Patrick S., Thomas L., Timo S. (2009). Potential novel drug carriers for inner ear treatment: Hyperbranched polylysine and lipid nanocapsules. Nanomedicine.

[24] Mohammed F.A., Balaji K., Girilal M., Kalaichelvan P.T., Venkatesan R. (2009). Mycobased Synthesis of Silver Nanoparticles and Their Incorporation into Sodium Alginate Films for Vegetable and Fruit Preservation. J. Agric. Food Chem..

[25] Li Y.N., Ye Q.Q., Hou W.F., Zhang G.Q. (2018). Development of antibacterial ε-polylysine/chitosan hybrid films and the effect on citrus. Int. J. Biol. Macromol..

[26] Sun J., Ren R., Yao L., Li J., Tong L., Yuan J., Wang D. (2024). Effect of Combined Chitosan and Hyperbranched Poly-L-Lysine Based Coating on Prolonging the Shelf Life of Oyster Mushroom (*Pleurotus ostreatus*). Foods.

[27] Hodges D.M., Toivonen P.M.A. (2008). Quality of fresh-cut fruits and vegetables as affected by exposure to abiotic stress. Postharvest Biol. Technol..

[28] Ewa R., Kadir S., Fatih M.A. (2022). Preservation effects evaluated using innovative models developed by machine learning on cucumber flesh. Eur. Food Res. Technol..

[29] Li X.Y., Liu Y.H., Gao Z.J., Xie Y.K., Wang H. (2022). Relative humidity control during shiitake mushroom (Lentinus edodes) hot air drying based on appearance quality. J. Food Process Eng..

[30] (2016). Microbiological Examination of Food Hygiene—General.

[31] (2016). Determination of Total Microbial Colonies in Food.

[32] (2016). National Food Safety Standard—Determination of Ascorbic Acid in Foods.

[33] Gao X., Ohlander M., Jeppsson N., Björk L., Trajkovski V. (2000). Changes in antioxidant effects and their relationship to phytonutrients in fruits of sea buckthorn (*Hippophae rhamnoides* L.) during maturation. J. Agric. Food Chem..

[34] Li N., Chen F.M., Cui F.J., Sun W.J., Zhang J.S., Qian L.S., Yang Y., Wu D., Dong Y., Jiang J.X. (2017). Improved postharvest quality and respiratory activity of straw mushroom (*Volvariella volvacea*) with ultrasound treatment and controlled relative humidity. Sci. Hortic..

[35] Gómez-López V.M., Devlieghere F., Ragaert P., Debevere J. (2007). Shelf-life extension of minimally processed carrots by gaseous chlorine dioxide. Int. J. Food Microbiol..

[36] Fan K., Zhang M., Guo C.F., Devahastin S. (2021). Laser-Induced Microporous Modified Atmosphere Packaging and Chitosan Carbon-Dot Coating as a Novel Combined Preservation Method for Fresh-Cut Cucumber. Food Bioprocess. Technol..

[37] Olawuyi I.F., Jin P.J., Jun L.J., Young L.W. (2019). Combined effect of chitosan coating and modified atmosphere packaging on fresh-cut cucumber. Nutr. Food Sci..

[38] Zhang L.P., Xie J., Wang T., Xiong Q. (2013). Study of Physicochemical Properties of Chinese Small Cabbage(*Brassica chinensis* L.) Stored at Four Temperatures. Adv. Mater. Res..

[39] Fan K., Zhang M., Fan D.C., Jiang F.J. (2019). Effect of carbon dots with chitosan coating on microorganisms and storage quality of modified-atmosphere-packaged fresh-cut cucumber. J. Sci. Food Agric..

[40] Pesis E. (2005). The role of the anaerobic metabolites, acetaldehyde and ethanol, in fruit ripening, enhancement of fruit quality and fruit deterioration. Postharvest Biol. Technol..

[41] Yuan G.F., Wang S., Gao W.F., Chen X.E. (2023). Effects of chitosan with different molecular weights on storage quality and fungi inhibition of mini-cucumber. Food Control.

[42] Hu Y.H., Chen C.M., Xu L., Cui Y., Yu X.Y., Gao H.J., Wang Q., Liu K., Shi Y., Chen Q.X. (2015). Postharvest application of 4-methoxy cinnamic acid for extending the shelf life of mushroom (Agaricus bisporus). Postharvest Biol. Technol..

[43] Khademi O., Ashtari M., Razavi F. (2019). Effects of salicylic acid and ultrasound treatments on chilling injury control and quality preservation in banana fruit during cold storage. Sci. Hortic..

